# BlockPres: A Novel Blockchain-Based Incentive Mechanism to Mitigate Inequalities for Prescription Management System

**DOI:** 10.3390/s21155035

**Published:** 2021-07-25

**Authors:** Alan Litchfield, Arshad Khan

**Affiliations:** Service and Cloud Computing Research Lab, Auckland University of Technology, Auckland 1010, New Zealand; arshad.khan@aut.ac.nz

**Keywords:** blockchain, Healthcare Information System, prescription management system, accessibility, incentive mechanism, healthcare inequality

## Abstract

The study presents a blockchain-based incentive mechanism intended to encourage those in underserved communities to engage with healthcare services. The smart healthcare system, which is the result of the amalgamation of advanced technologies, has emerged recently and is increasingly seen as essential to meet the needs of modern society. An important part of the healthcare system is the prescription management system, but studies show that prescription affordability and accessibility play a part in creating unequal access for underserved communities. This is a form of unequal access that results in those living in underserved communities to become disengaged from accessing healthcare services. In New Zealand, the prescription management system plays a crucial role and this study seeks to address the issue by presenting the BlockPres framework, which uses a novel incentive mechanism to encourage patients to participate and engage with services in order to be rewarded. The blockchain attribute of immutability in BlockPres enhances equality and participation by providing sophisticated authorisation and authentication capabilities for healthcare providers and patients. BlockPres empowers the patient by assigning ownership or control of some patient information to the patient. A simulation is carried out using the Ethereum blockchain and the evaluation of successful transaction completion and superficial performance assessment demonstrates that the blockchain would be sufficient to cope with the needs of a prescription management system. Furthermore, for the simulation, a BlockPres Smart Contract is developed using solidity and implemented in Remix. The Ropsten network is used as the simulation environment and the initial results show that the proposed incentive mechanism mitigates unequal access.

## 1. Introduction

This study uses blockchain technology to enable an incentive mechanism to encourage and attract patients to participate and engage with a Prescription Management System (PMS) and to receive some form of compensation that may be exchanged for the cost of future prescriptions, doctor visits and so on. The paper presents the blockchain based PMS, BlockPres, that addresses issues outlined below and seeks to resolve lack of trust and unfortunate perceptions of the healthcare system among underserved communities in New Zealand.

Healthcare data management is a process intended to improve patient outcomes through the treatment process, efficient tracking of disease, identifying causal relationships in the appearance of diseases, to guide the production of medicines, and to provide pathways for disease prevention. In general terms, a manual system collects data from patient interactions and visits and the data are stored in the patient record. The emergence of Electronic Health Records (EHR) in the Healthcare Information System (HIS) has enabled more efficient sharing of data within and between healthcare organisations, medical drug manufacturers, pharmacists, medical insurance providers, researchers and patients [1]. An HIS consists of a range of information systems, including the PMS [2].

This study focuses on the New Zealand PMS and issues related to underserved communities, in particular, the Māori and Pasifika. Research indicates that under served communities experience unequal access to HIS and, given the broad range of systems that people may interact with, this study specifically addresses the PMS. There are indications that as people experience unequal access, they also become disengaged from the services provided. Factors resulting in patients becoming disengaged include, amongst others, a lack of trust, the cost of treatment and prescriptions and the distance to the healthcare provider plus the cost of transport [2,3,4].

The process of storing (such as historical prescriptions) and transferring patient data across multiple entities is complicated by a heterogeneous and poorly integrated information systems environment [2]. This is not a new problem and there have been attempts at solving this problem before, for example, [3]. The PMS environment is crucial since the responsibility for maintaining an accurate prescription record is shared across healthcare providers. In this study, the proposed system is addressed with blockchain technology.

Blockchain technology is a distributed ledger that maintains its transaction history across a decentralised network of nodes that retain copies of the ledger. The blockchain is updated using a one of a large range of consensus based protocols. In that sense, there is an expectation that there is no trust in the community but that all contributing members have trust in the efficacy of the consensus protocol. The ledger then provides immutable transaction logs and they are typically open to public scrutiny [5].

The main contributions of this study are that by applying a blockchain technology solution, we hope to attain the following:

1. An incentive mechanism to encourage underserved communities to participate in the delivery of healthcare services;
2. A framework that seeks to change patient behaviours by altering perceptions about inequality or unequal access and to encourage underserved communities to participate and use the healthcare system;
3. A system that provides a patient-centric approach where patients control parts of their record and the authorisation process.

The structure of the paper is as follow: Section 2 provides a survey of related work. Section 3 describes the problem being addressed in this study. Section 4 briefly describes the research method. In order to overcome the issue of inequalities, Section 5 presents the conceptual BlockPres PMS. Section 6 provides a description of the incentivisation scheme. Section 7 describes the application of cryptographic keys in BlockPres and, in Section 8, the protocols used in the system are described. Section 9 presents the experiments in which the BlockPres model is evaluated and the effectiveness for its prototype development is presented. The conclusion and directions for future work are presented in Section 10.

## 2. Related Work and Theoretical Foundation

In the HIS environment, sensitive private data are the norm and are distributed between healthcare providers as a matter of course [2]. What is of concern to providers are limited data accessibility and incomplete data where patients may suffer actual harm in HIS [4,6]. The data need to be delivered in a timely fashion and in a form that is compatible with the receiver [6,7]. For example, timely access to patient data is essential to ensure continuous and correct treatment [8] and the presentation of patient data in transfers between healthcare providers or treatment facilities [9]. This raises two issues, which include what data providers require to share the data and whether systems are in place to share the data seamlessly. To define relationships between providers, the blockchain solution MedRec applies smart contracts where relevant data are preserved on the ledger [10]. MedRec also empowers the patient to reject or accept a patient–provider relationship.

Overall, any improvement observed in the management of patient records should result in greater control of patients’ personal records [11]. The security of data held by providers is important and, thus, measures are required to detect and prevent intrusion [9]. Where poorly defined or managed access control policies exist, poor standards are applied to authentication methods, credential sharing or weak passwords are allowed; in this case, breaches can and do result [12]. Attempts to address these issues have been made and they include the two protocols for Distributed Ledger Technologies (DLT) to improve IEEE 8.02.15.6 and to establish secure links for mobile devices with unbalanced computational requirements and the other to distribute healthcare data among Pervasive Social Network devices [13]. An alternative approach is to provide the same level of security but with less overhead which renders it more challenging to discern access control privileges through the application of smart contracts [14] or with cryptographic signatures [15]. DLTs can also offer a decentralised and consensus-based approach to privacy, security measures and patient data tracking. In addition, if there is no single point of failure in a system, then it may be argued that the deployment of a DLT with its associated redundancy provides greater likelihood of the maintenance of data integrity [6].

The blockchain’s implied immutability means that once records are appended to the chain, they cannot be altered easily [13]. While this presents advantages such as preventing unauthorised changes, it also means that errors may be more challenging to correct in subsequent additions. In such a scenario, an error in data requires a new record to be appended and an interface that reads and reports on the DLT must retrieve the latest entry. Thus, to minimise error rates, the design of the HIS is critical for patient safety. Factors to consider when improving HIS include naturalness, consistency, error prevention, minimisation of cognitive load, interaction efficiencies, feedback mechanisms, effective use of language and customisability or flexibility [3]. Thus, these improvements provide caregivers, healthcare providers, clinicians and technicians more time for individual patients [16].

Instances exist where the quality or veracity of data may only be assured if there is third-party notarisation of a smart contract. For example, when a biomedical database is queried, the enquirer may need assurance that the data are valid [8]. Across the range of health services, the volume of data in HIS is enormous, complex and heterogeneous. Furthermore, the number of dependent and independent HIS that are poorly integrated, the constant updates to existing data, inconsistent data representation and data structures, missing and incomplete data and the difficulty in finding the required answers in large data sets returned from queries [17] renders knowledge discovery difficult and expensive. The DLT provides the opportunity to develop an enterprise bus or a searchable index [8]. Moreover, DLT applications in the areas of supply chain management and provenance tracking have been developed and this is particularly useful in the tracking of drugs with a chain of custody and permits the ability to trace where drugs have been or come from and provenance tracking permits the tracing of counterfeit drugs that may have found a path into the supply chain [18]. Another example of how accurate returns on queries facilitate knowledge acquisition from data is pandemic or epidemic identification by isolating, discovering and driving change for environmental conditions that impact public health [19].

Where patients pay directly for healthcare services and insurance may be used to reimburse costs, the insurer needs assurance that costs are accurate and not inflated [20]. Incorrect billing may be a result of inconsistencies in recorded data, inaccuracies in inpatient medical histories and patient information not shared with healthcare providers and stakeholders [8]; in this case, a DLT can provide transparency and accuracy in billing [21]. Furthermore, if a patient takes ownership of their health record, then the patient should be able to exercise greater control over their expenses and make decisions based on the financial impact of healthcare costs [22]. Smart contracts can be applied to a patient’s healthcare record as a means of alerting providers of treatments or tests that have already been undertaken or additional tests and treatments that may not be necessary [19].

Apart from those examples above, other examples that illustrate the development and implementation of blockchain based systems that use smart contracts include a framework to store patient’s data securely, where patient data are stored on a secure cloud and are accessible upon the authorisation of users [23]. The smart contract can also be used for secure communication between patients and professionals and can notify professionals about patient activities during their stay at hospitals. MeDShare is a blockchain-based system for medical data sharing and provides auditing, data provenance and the control and monitoring of patient data stored in cloud repositories [24]. Patient monitoring has also been proposed [25]. Another example provides an electronic healthcare system using blockchain for a wireless body area network. The system used wireless body sensors to collect patient data and sends it to the blockchain network [26]. To exchange data between healthcare providers, another solution uses magnetic resonance images as a formal method to capture patient information [27].

None of the studies found propose to solve healthcare access issues relative to underserved communities. Studies tend to focus on technological frameworks or variations on billing and security systems; therefore, this study addresses equality, engagement and incentives for underserved communities by using blockchain technology in the design of a PMS.

## 3. Problem Definition

A range of factors may serve to identify a group as being an underserved community. In addition to the factors that have been identified, we note that members of underserved communities tend to become disengaged from healthcare services. In this section, we describe some of the factors that are related to this study and we define the problem area. However, communities in other cultural or geographic regions may identify different sets of factors.

A common factor that affects a patient’s perception and trust towards the system is a personal belief held that the healthcare system treats the patient unfairly or does not provide equal access to services. This results in the patient becoming disengaged from the healthcare system. This may be because the patient sees the public health system as hostile and alienating and that may be a consequence of the patient’s inability to pay the cost of treatment and prescriptions, a patient living in a rural area may experience high transport costs or difficulties in getting to a hospital or clinic, the inability to take leave from work or personal beliefs that run counter to established medical practices [28].

Over the past several decades, developments in technology have seen a rapid growth of digital devices and technologies to improve HIS [29]. However, groups that are considered to be underserved have emerged over the same period [30] and, in this region, underserved Māori and Pasifika groups with limited access to digital infrastructure have been identified [28]. Factors that typify these groups, amongst others, are long-term medical or disability issues and cultural or language barriers. Additional problems are that underserved communities believe that the system treats them differently from what may be described as a “served” community. Consequently, lack of trust emerges and the unwillingness to make use of healthcare systems available results. In all these cases, social impacts arise when an unequal level of access to digital platforms exists, which in the current environment can result in an unequal level of healthcare delivery [31].

The level of health and well-being amongst Māori populations is reasonably well documented ([31,32], for example). Studies repeatedly show that there exists wellness gaps between Māori and non-Māori and that these include lifestyle factors, levels of existing health conditions and the life expectancy gap is more than eight years between the groups. Rates of smoking tobacco amongst the Māori is 50% higher than non-Māori, resulting in a mortality rate of up to 10%. Even though successive governments have made promises to reduce inequities over the past decade, the problem continues to increase and healthcare systems fail to overcome inequity problems in all population groups [33]. In addition, while recent developments and reforms in the delivery of healthcare services have been made, the problem still exists and accessibility remains, which contributes to inefficiencies and inequities.

## 4. Methodology

In order to design and develop a framework as a solution to the research problem described above, the Design Science Research (DSR) methodology is adopted [34]. This methodology is used because it allows the extension of boundaries in human and organisational capabilities by creating new and innovative artefacts. For this study, the DSR process is comprised of the following three phases: problem identification, solution design and evaluation. Each phase comprises different steps [35,36]. The design process incorporates the definition of the problem statement and the design of a framework as a conceptual model, which is then refined as a logical model that is evaluated in an iterative process of instantiations to determine the quality of the logical models. The primary purpose of this process is to produce an effective system in the form of blockchain-based PMS.

## 5. BlockPres Framework

In this section, the BlockPres framework is presented. Table 1 provides notations and descriptions used in the framework and details that follow. Since the overall BlockPres framework is extensive, this paper will only address those related to hospital and GP generated prescriptions.

### 5.1. System Components

This section provides descriptions of the entities or system participants involved in BlockPres. In order to enhance the efficiency of patient treatment and to build trust in the system, healthcare providers want to share patient healthcare records with peers. The framework consists of system components that include the New Zealand Ministry of Health (MOH), healthNZ and healthcare providers such as doctors, nurses, hospitals and pharmacies [37].

**MOH**  The government agency that regulates healthcare systems running in New Zealand. All healthcare providers and pharmacies are registered with MOH. In BlockPres, MOH generates parameters for healthcare providers and provides a unique public key.
**HealthNZ**  Exists in each district to control and manage healthcare providers and pharmacies. HealthNZ is responsible for the integration of services provided to healthcare providers and patients.
**Healthcare providers**  Medical service providers who provide medical services to patients. The healthcare providers consist of medical staff such as doctors and nurses. Medical staff have access to local computer systems and HIS. In BlockPres, the doctor enters patient data and the data are copied to a hospital server. The local database maintains a private blockchain that verifies incoming blocks. The doctor broadcasts unique keywords generated from individual patient records to a public blockchain. Patient registration and prescription records are stored locally. When a healthcare provider receives a request from another healthcare provider to a access patient record, the public blockchain provides authentication of the entity.
**User**  Users are patients in the system and are the primary entities in BlockPres. Patients can either register online to see the doctor or visit in person to obtain an appointment. Each user obtains a public key called a National Health Index (NHI) to interact with the healthcare provider or doctor. The specific NHI number is evidence that the patient receives the treatment and the doctor then generates the record.

### 5.2. System Design and Workflow

**Figure 1:**
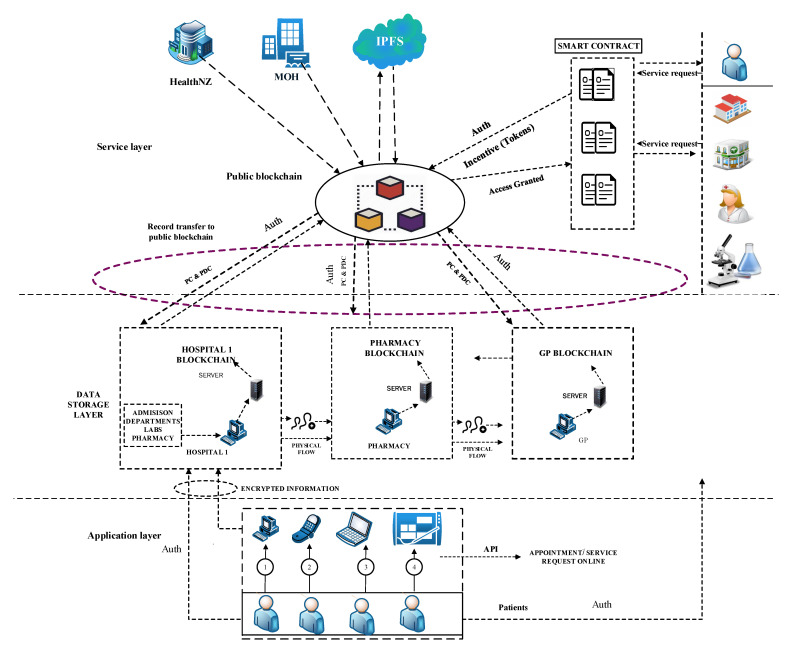
BlockPres Framework.

The BlockPres framework and its workflow (Figure 1) is divided into the following three sections: the application layer, data storage layer and service layer. The figure describes the patient’s registration and prescription process from a healthcare provider to a pharmacy and how patients obtain incentives; registration provides permission to their records. The capability to store data on blockchain and IPFS is also included.

### 5.3. BlockPres Application Layers

The application layer provides an Application Programming Interface (API) for the system participants. The system participants are denoted by the following.
Patients$PT_{K}\left(PT_{1},PT_{2},PT_{3},\cdots PT_{m}\right)$Doctors$DT_{K}\left(DT_{1},DT_{2},DT_{3},\cdots DT_{m}\right)$Nurses$N_{K}\left(N_{1},N_{2},N_{3},\cdots N_{m}\right)$Hospitals$H_{K}\left(H_{1},H_{2},H_{3},\cdots H_{m}\right)$Pharmacies $PH_{K}\left(PH_{1},PH_{2},PH_{3},\cdots PH_{m}\right)$

**Figure 2:**
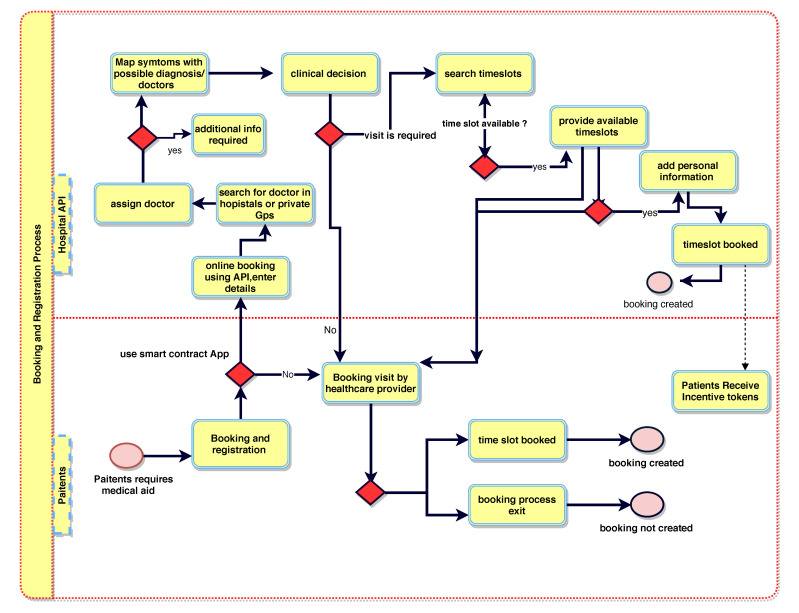
BlockPres booking and registration process.

**Figure 3:**
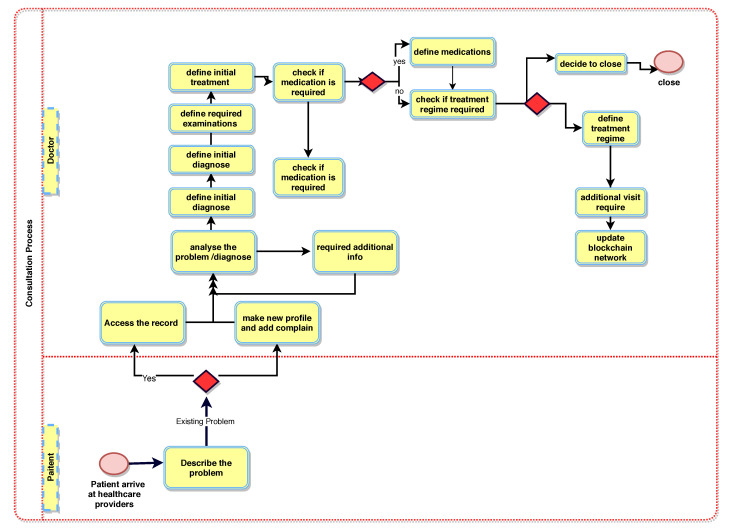
BlockPres consultation process.

**Figure 4:**
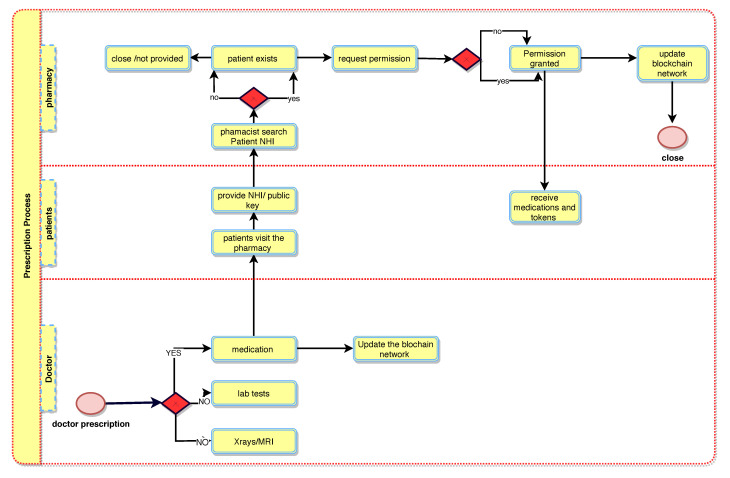
BlockPres prescription process.

Figure 2 illustrates the booking and registration process. When the patient, $PT_{K}$, is registered with BlockPres, they are provided with public and private identifiers (IDs). The IDs allow for further interactions on the system and the authorisation of events as they occur. The patient, $PT_{K}$, obtains an appointment online by using the API or he can travel straight to the hospital. Figure 3 illustrates the consultation process and Figure 4 presents the prescription process of patients traveling from the healthcare provider to the pharmacy.

### 5.4. Data Storage Layer of Proposed Framework

Participant records are stored at the data storage layer. When a patient, $PT_{K}$, visits a hospital $H_{K}$ or general practitioner $GPS_{K}$ for service, the $H_{K}$ administrator registers the patient’s presentation information; otherwise, the patient $PT_{K}$ registers online with a device and using the API (Figure 2).

Algorithm 1 presents the patient registration to the diagnostic service process. For any case, a Registry Contract, RC (part of the Smart Contract, SC), is required to be signed. The patient, $PT_{K}$, provides personal information and presentation information in the RC. During the consult, the doctor, $DT_{K}$, assesses the patient, $PT_{K}$, and, where it is required, prescribes treatment or makes a request for further testing (Figure 3). When a doctor, $DT_{K}$, prescribes medication or makes a request for tests (Figure 4), a transaction will result and consists of the IDs for the doctor ($DT_{K}$), patient ($PT_{K}$), details of medications or tests, dosage instructions and a timestamp.
 **Algorithm 1:** Patient visits the Hospital/GP for a specific problem 
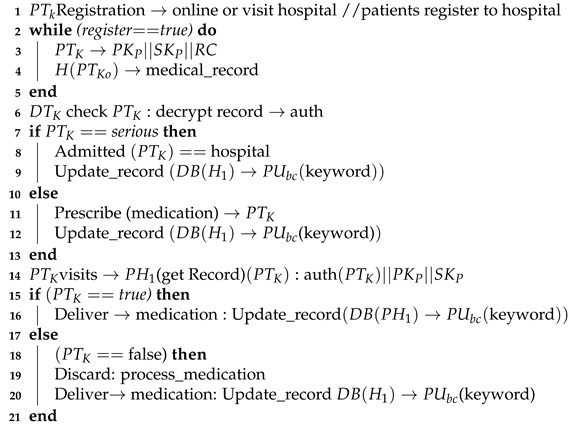


When the transaction is assembled, the record is stored in a transaction pool to be added to a block in a DB for the hospital $H_{K}$. When the pooled transactions have been validated, they are added to a public ledger. Note that no patient data are added at this point. The public ledger can only contain a record of the smart contract’s existence.

**Figure 5:**
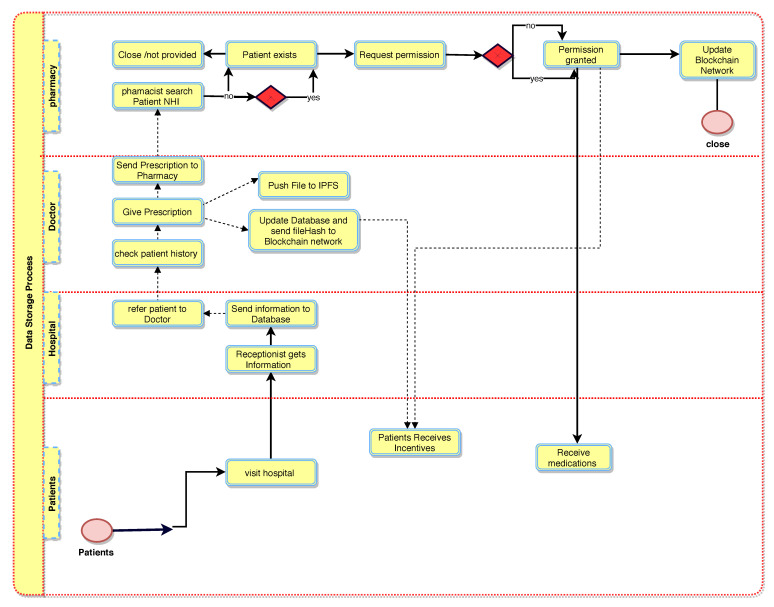
BlockPres data storage process.

A patient, $PT_{K}$, may then visit a pharmacy or other healthcare providers (Algorithms 2 and 3) and provide the public key to grant access to the prescription transaction (Figure 5). The pharmacy will use the patient’s ($PT_{K}$) private key to decrypt the transaction. The service agent cannot have access to the key itself because this is an automated process. A record of the completed prescription is created, which is encrypted using the patient’s ($PT_{K}$) private key and stored on a single chain and then submitted to the public chain.
 **Algorithm 2:** Accessing patient’s record from a different healthcare provider 
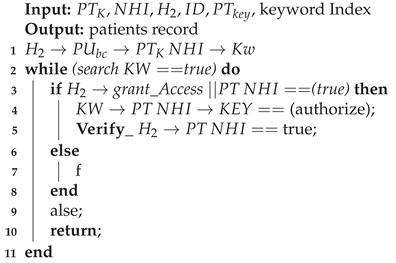


 **Algorithm 3:** Patient’s visits to a different hospital 
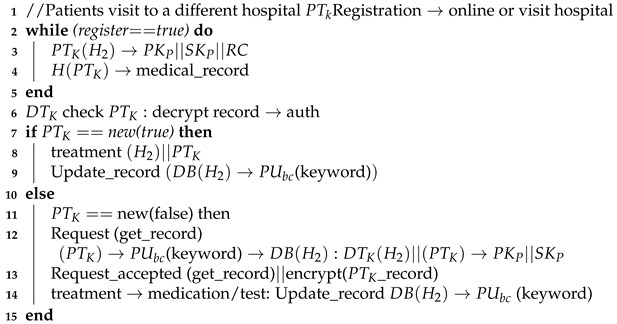


### 5.5. BlockPres Service Layer

**Figure 6:**
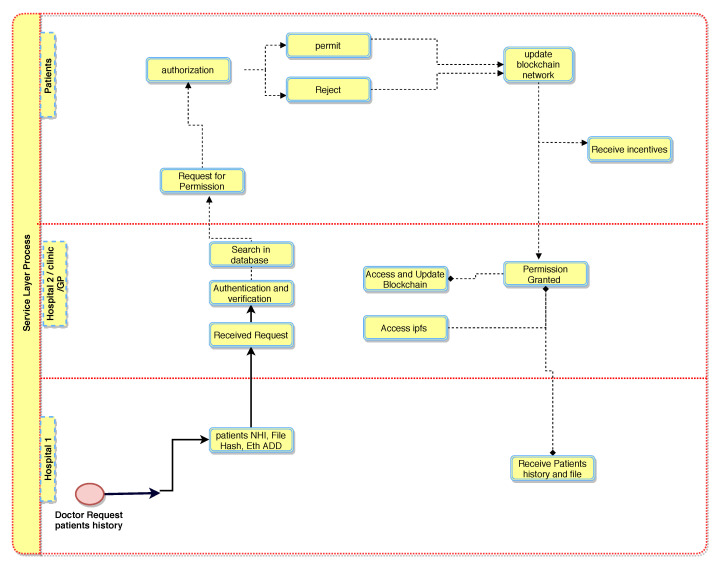
BlockPres service layer processes.

In this layer, data are stored in a second layer by healthcare providers and uploaded to a public blockchain, PUbc, to provide services to the healthcare provider (Figure 6). The lower layer, $H_{1}$, holds encrypted data from patients and information is stored in a healthcare provider database, DB, and then the data are broadcasted to the decentralised and distributed network. The selected systems are responsible for verifying the blocks of data before forwarding them to the public blockchain, PUbc. In this phase, the patient’s ($PT_{K}$) record is stored in a public blockchain (PUbc), for example, the InterPlanetary File System (IPFS) [38,39,40]. IPFS is a Distributed File System (DFS) that operates as an alternative to the Domain Name System (DNS) that currently dominates the Internet. IPFS promises to distribute the World Wide Web and render it more efficient. IPFS is appropriate for this solution because it can store large files and the data can be retrieved using keywords or a hash of the related content [41,42,43].

**Figure 7:**
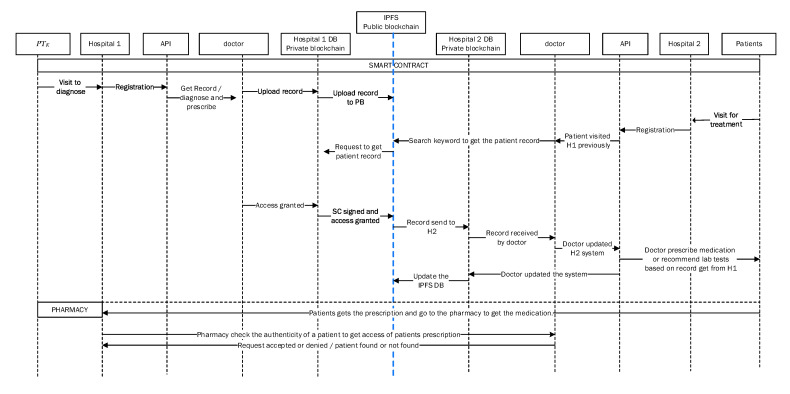
BlockPres transaction processes.

Figure 7 illustrates communication and transaction processes between entities in the healthcare system and Algorithm 3 describes the process of obtaining patient records from various hospitals. When a third-party provider such as a pharmacy needs to access a patient record, permission is obtained from the patient ($PT_{K}$). The healthcare provider sends a service request to the public blockchain, PUbc, and then to a healthcare provider, Hp. The process of accessing the data is secured by public encryption with search keywords [42,44]. The healthcare provider must sign the patient’s $PT_{K}$RC on the SC and then sign a Permission Contract to access the patient’s $PT_{K}$ data. In order to access the patient’s ($PT_{K}$) data, a Permission Contract (PC) is used to sign an agreement between healthcare providers and to obtain confirmation from the patient ($PT_{K}$) before sharing their data with other providers. Moreover, this phase includes an incentive mechanism (Algorithm 4) to encourage patients to use the healthcare system and to behave honestly when sharing medical records with healthcare providers. In return, patients will obtain incentives as Ethereum tokens by using the ERC-20 protocol [45,46]. Patients can use the tokens earned wherever they may be redeemed to obtain discounts on healthcare charges, to purchase coffee in a coffee shop, to purchase apparel from clothing stores and so on.

The BlockPres framework provides critical functions. The first function is to enhance equality across the PMS. Secondly, the network is decentralized and thus records are distributed since the blockchain is highly redundant [47,48]. Every network node receives an updated copy of all records [49,50]. Thirdly, the system provides integration, which enhances integrity and trust. The fourth function is to provide an incentive mechanism to encourage patients to participate and use the healthcare system and to behave honestly to obtain rewards in tokens.
 **Algorithm 4:** Patient Obtaining Incentives 
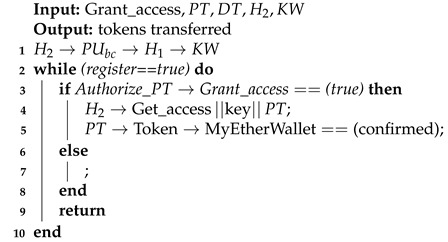


## 6. Incentive Mechanism to Mitigate Unequal Access

In this section, an incentivisation mechanism to mitigate negative effects of unequal access to healthcare services is described. Perceptions that prevent engagement in the fulfilment of prescriptions may be overcome if patients are encouraged to participate through incentivisation. In this study, a system that incorporates cryptocurrencies might show positive benefits if an incentivisation scheme was introduced to the prescription fulfilment process. The incentive is to earn tokens as a reward for prescriptions that are successfully filled. The tokens may be redeemed for health services, products, services and so on. It is also possible that the patient can send their earned tokens to others to help them obtain additional services.

**Figure 8:**
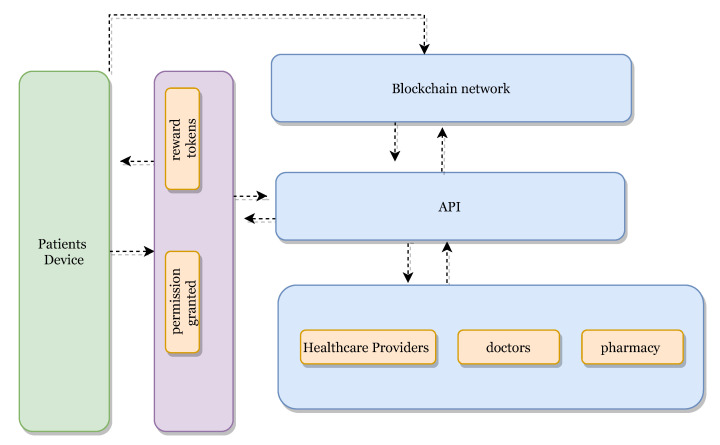
BlockPres incentive mechanism.

The incentive platform is built on a cryptocurrency blockchain with a specifically designed incentivisation protocol. Algorithm 4 provides an incentive or reward for patients that provide access to their records. When a patient signs up for the service using the API (Figure 8), the patient’s account creates a unique address for authorisation and identification. A patient crypto wallet is installed which enables the patient to receive rewards from the system. It is also necessary to link the system to appointment bookings and prescription repeats. The cases below show how the incentive is accounted for with respect to the patient’s wallet.

**Case 1**  Whenever patients visit a healthcare provider or doctor for treatment and register with healthcare providers, the patient receives a reward (token). The workflow of the incentivisation process is illustrated in Figure 6.
**Case 2**  When a patient is issued a prescription from the doctor and then visits a pharmacy to obtain the medication, the pharmacy enters the patient NHI to obtain the prescription. The access request alerts the doctor for authorisation and, at the same time, the patient receives an authorisation request. The patient receives a reward for providing authorisation for access with respect to obtaining prescription from the pharmacy.
**Case 3**  Whenever the patient visits other healthcare providers or doctors, for example, in cases of emergency, the doctor accesses the patient’s previous record or history of treatments and prescriptions. The doctor sends a request to the patient healthcare provider for granting access to the patient’s record. In this case, the patient will receive permission requests from their healthcare provider doctor:“the provider, ABC, needs to access your record, do you give permission?” Once the patient’s permission is obtained, the patient will receive a reward.
**Case 4**  When the healthcare provider shares a patient record for any purpose with any other healthcare provider, doctor and organisation, the patient will receive incentives (tokens) for permission to access the record.

## 7. Utilisation of Cryptographic Keys in BlockPres

In this section, the utilisation of cryptographic keys is described. Cryptographic keys play a significant role to ensure data privacy [51,52]. Public/private key pairs are used to provide $PT_{K}$ transaction confidentiality when the record traverses untrusted channels [53,54]. In BlockPres, there are multiple entities $PT_{K}$, $DT_{K}$, $N_{K}$, AD, $PH_{K}$ and $NS_{K}$ and thus the system creates keys for each entity using a cryptographic method called El Gamal [55,56,57]. The key pair of an entity is symbolised by $PK_{k}$ and $SK_{k}$, where $PK_{k}$ is a public key of an entity and $SK_{k}$ is a private or secret key of an entity. Moreover, the $SK_{k}$ must be kept secret by the entity, while $PK_{k}$ can be distributed among healthcare providers and other entities in the system. Therefore, the public key set is $PK_{k}=\left(PK_{1},PK_{2},PK_{3},PK_{4},\cdots PK_{k}\right)$. The secret or private key relation is established between entity A and B by using a secure algorithm (for example, AES) [57]. A Diffie–Hellman key exchange mechanism is responsible for establishing keys before communication occurs between A and B and it is only known to the entities communicating with one another [58,59,60]. The keys are required to ensure the integrity, security and authenticity of the transactions when both entities generate transactions [38,52].

### 7.1. Transactions Patterns

In BlockPres, a set of attributes is defined as a transaction related to the $PT_{K}$ prescription record and information inside the record is encrypted with SK between the sender and receiver. In this case, the sender and receiver can be $PT_{K}$, $DT_{K}$, healthcare providers and vice versa. There are three types of the transactions described in the following sections: Genesis transaction ($Tx_{Gen}$), DB transaction ($TX_{DB}$) and $PU_{bc}$ transaction ($TX_{PU_{bc}}$).

### 7.2. Genesis Transaction

Genesis transaction ($Tx_{Gen}$) (Equations (1) and (2)) creates the first hash in a new blockchain. Initially, the transaction is created when the $PT_{K}$ registers and is stored in a hospital database. The DB stores data from the connected department in a hospital, for example, a surgical dept where the following is the case:
$Tx_{Gen}$  is a genesis transaction created by any user in the system;txid  is a transaction ID;PTid  is a patient ID;SKP  is a secret key;PKP  is a public key;SC  is a smart contract;DB  is a private database;Signs1,s2,s3,s4,⋯sn  is a message signed by the patient/doctor using a private key which contains attributes related patients medical record.

(1) $$Tx_{Gen}=Reg\left(\left[Fname,Lname,Add,\right],SKP,PKP,SC,Sign\right)$$

(2) $$Tx_{Gen}=\epsilon nc\left(\left[txid,PTid,Sign\left(s_{1},s_{2},s_{3},s_{4},\cdots s_{n},PKP\right)\right],SKP,DB\right)$$

Equation (2) is the encrypted transaction created by the users in using a private key.

### 7.3. Local Database Transaction

In order to store the prescription record, $PT_{K}$, in the hospital DB and for validation, this transaction (Equation (3)) is created by the healthcare provider: $DT_{K}$, $N_{K}$ and administration. The transaction can be represented as a tuple where, in addition to the previous variables, the following are included:
$Tx_{DB}$  is a DB transaction created by any user in the system;$DT_{K}$  is a Doctor ID;SKd  is a doctor secret key;PKd  is a doctor public key.

(3) $$\begin{array}{cc} TxDB= & \epsilon nc(\left[txid,PTid,Sign\left(s1,s2,s3,s4,\cdots sn,PKP\right)\right],SKP,[DT_{K},SKd,PKd,\\ \; & Sign(s1,s2,s3,s4,\cdots sn,DB)]) \end{array}$$

Equation (3) is the encrypted transaction created by the healthcare providers to store the patient record using the private key.

### 7.4. Public Blockchain Transaction

Healthcare providers generate this transaction (Equation (4)) to upload patient records as keywords to IPFS, which works as a PUbc in the system. The transaction accesses the record at a healthcare provider if and only if the patient has a prescription record. This transaction is represented as a tuple below.
TxPUbc  is a public blockchain transaction;Kw  is a keywords search by healthcare providers in a PUbc;PRd  is a patient record.

(4) $$TxPUbc=\epsilon nc(\left[txid,PTid,PKP\right],\left[DT_{2}ID,PKd,Sign,kw,SC,DB\right],PUbc)$$

In the transaction above, $H_{2}$ sends a request to $H_{1}$DB from PUbc to access a specific patient record. By signing SC using a public and private key, the transaction in Equation (5) represents the reply from $H_{1}$, providing the patient record and allowing $H_{2}$ access to the patient record.

(5) $$TxPUbc=\epsilon nc(\left[txid,PTid,PKP\right],\left[DT_{2}ID,PKd,Sign,kw,SC,DB,PRd\right],PUbc)$$

## 8. BlockPres Protocol Description

In this section, the protocols applied in BlockPres are described. The protocol comprises the three following phases: Setup, User Registration (which includes Encryption and Decryption) and Incentive Mechanism.

### 8.1. Phase 1: Setup (λ)

The hospital, $H_{2}$, runs the setup algorithm and takes the security parameter (λ) as input. The output of the system setup parameter is the public key (PK) and master key (MK). Then $H_{2}$ publishes the public key on media or in a database. $H_{1}$ encrypts the MK and embeds it into the transaction. $H_{1}$ also runs the smart contract on the blockchain. The smart contract provides access to DT or $H_{2}$ as encrypted indexes stored on the blockchain network. When $H_{2}$ sends a request for registration to $H_{1}$, $H_{2}$ first needs to check the identity of the $H_{1}$. After confirming the $H_{1}$, $H_{2}$ assigns an attribute set *S* and adds the $H_{1}$ Ethereum account address to the smart contract, whereupon $H_{2}$ generates SK.

### 8.2. Phase 2: User Registration

This algorithm, run by $H_{1}$, takes the input of PT, NHI and DT attributes *S*. The output will be SK. The DT private key is encrypted and secured using the AES algorithm and attached to the Ethereum account. The encrypted key is generated using the Diffie–Hellman key exchange protocol and $H_{1}$ sends the PT transaction ID and smart contract non-repudiation signature through a secured channel.

#### 8.2.1. Encryption

The encryption algorithm runs by $H_{K}$ and consists of the following algorithms.

**EncryptingFile**  This algorithm takes input in a shared file and provides as output the ciphertext CT, *K* and kw. The $H_{K}$ selects a set of keywords, kw, from the shared file, key *K* from AES keyspace and uploads the CT to IPFS.
**KeyEncryption**  This algorithm takes input PK as the public parameter, *K* as the file encryption key and the location of the file. It provides an output of ciphertext CT. $H_{K}$ uses *K* to encrypt ciphertext and the location and uses the AES algorithm to encrypt file key *K*. The algorithm uses public parameters to encrypt *K* and the ciphertext. $H_{K}$ randomly selects the AES key, *K*, to encrypt CT and embeds it into the Ethereum transaction.
**IndexGen**  To access or share a file, this algorithm is run by either DT or $H_{K}$. It takes input a keyword, kw, and PTNHI. The output of this algorithm is a keyword index based on PTNHI from the smart contract initiated by both parties.

#### 8.2.2. Decryption

This algorithm is run by $H_{K}$ to access a PT record or file. It takes the file location in CT, AES encrypted keys *K*, DT and the secret key, SK, of the individual accessing the file. The output of this algorithm will be the original file. Based on the index keyword search, kw, of smart contracts, DT or $H_{K}$ reads the transactions from the Ethereum network. If the access policy meets the attribute *S*, then DT or $H_{K}$ decrypts the CT to obtain the original file from the IPFS.

### 8.3. Phase 3: Incentive Mechanism Process

This algorithm is run by $H_{1}$ to access PT records from $H_{2}$ as a request from PUbc. It takes the input of PT, NHI, CT, kw and PT(PK). $H_{1}$ sends an authorisation request to PT to grant access. In return, PT will receive a Tk from $H_{1}$ which is stored in a Wallet.

## 9. Experimental Results

This section presents an evaluation of the BlockPres framework and model. Section 9.1 details the simulation preparation, the environment and system specifications. Section 9.2 presents the preliminary simulation to validate the effectiveness of an instantiation of the model. When satisfied with the performance of the blockchain, an instantiation of BlockPres is presented in Section 9.3 and the effectiveness of the model is assessed.

### 9.1. System Specification and Simulation Environment

The evaluation uses the Ethereum network to perform a simulation of BlockPres. The Ethereum network provides more features than the bitcoin network [59,60], for example, the application of smart contracts and scripting through Solidity [38,61], that Ethereum consumes less computational power to validate transactions [62,63], Ethereum is able to validate more transactions per second than bitcoin [63] and the capability to build Decentralized Applications (DApps).

This simulation makes use of the Remix Integrated Development Environment (IDE), which uses the Solidity language to simulate the creation and use of Smart contracts [61]. In addition, Ganache is also used, which is a blockchain-based environment that provides virtual accounts that are linked to the Remix IDE and enables the execution of smart contracts. The ability for Ganache to produce unique IDs, the provision of mining processes to validate transactions and the ability to write the transactions to the blockchain provides the core functions in the simulation. Moreover, every virtual account has predefined amounts in the form of ether stored. Virtual accounts use these predefined ether amounts as a cryptocurrency [38]. The third important component is MetaMask, which is a browser extension that provides connectivity with Ganache and the Remix IDE [64]. The initial simulation is run on a local machine with the following specifications: Macbook Pro, HDD volume of 500 GB, 16 GB of RAM, CPU is a X64-based Intel processor running at 1.61 GHz and a 64-bit operating system.

### 9.2. Experiment 1: Preliminary Simulation for Blockchain Environment

To verify that a blockchain can process a sufficient number of service requests, two months of Ethereum transactions have been analysed [62]. The evaluation assesses the performance metrics block size, number of transactions per block, transactions per second, total number of transactions, median confirmation time and average block size.

**Figure 9:**
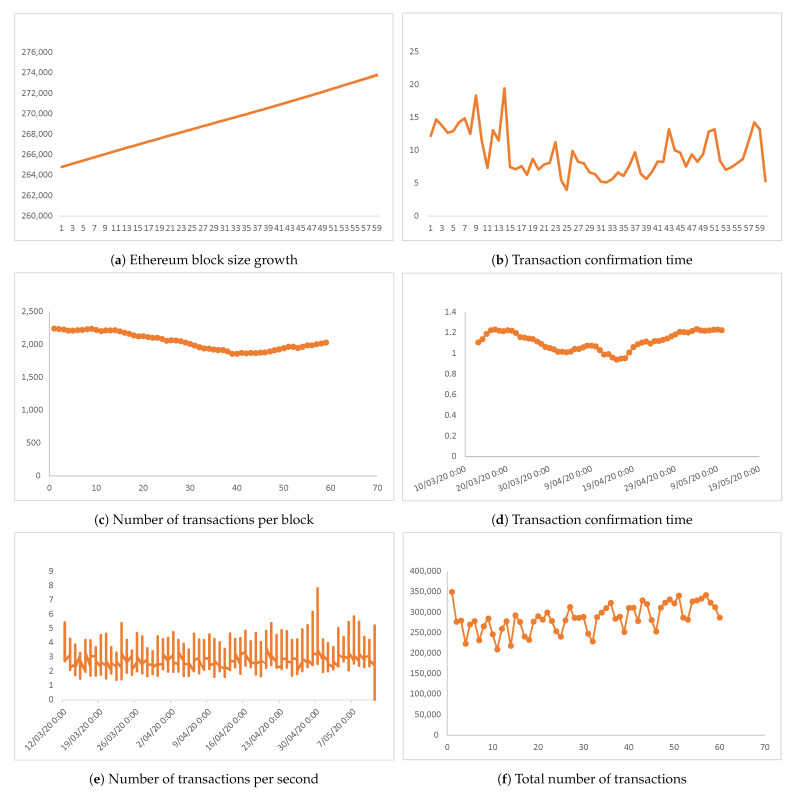
Raw Data of Experiment 1.

The data shown in Figure 9 illustrates Ethereum blockchain performance during the simulation. The blockchain grew at a more or less constant rate, at 2.686 GB per day (Figure 9a), but during that period the median confirmation time was less constant (Figure 9b), although the overall median confirmation time is 10.2 min per block. The number of unique transactions per block is 2200 (Figure 9c) and the average block size is 1.2 MB (Figure 9d). In terms of average speed, the Ethereum blockchain network executed six transactions per second (Figure 9e) with a total number of transactions processed per day of 360,000 (Figure 9f).

Consideration of the raw data allows a summary conclusion that Ethereum is sufficient to cope with the needs of BlockPres. This solution meets the basic requirements of BlockPres and satisfies the needs of storage and retrieval of patient records in a secure and trusted environment.

### 9.3. Experiment 2: Validation of the BlockPres model

Gas is a fundamental component of the Ethereum blockchain and its use impacts transaction speed and computational power [62,64]. There is an assumed difference in cost if Proof of Work (PoW) or Proof of Stake (PoS) are used. On the Ethereum network, each time a transaction is completed, a smart contract is executed and a cost is incurred measured as gas. The amount of gas consumed is the cost of mining blocks and sending them to the blockchain network. The unit of gas depends on the size of the block or smart contract complexity. For example, a simple transfer may use as much as 21,000 gas whereas a more complex transaction such as that seen in a complicated financial transaction could use more than 1,000,000 gas [65]. These issues have largely been resolved on the Ethereum ecosystem but the potential remains for excessive transaction costs.

Each unit of gas has a price referred to as the “gas price”. Gas prices are denoted in Gwei [66], where 1 ETH = $10^{1}8$ Gwei. Given a Gwei price of five, a 21,000 gas transaction would cost 21,000 × 5 = 105,000 Gwei. The transaction cost can be calculated by using Equation (6) [65].

(6) TotalCostGwei=GasUsed×GasCost

**Figure 10:**
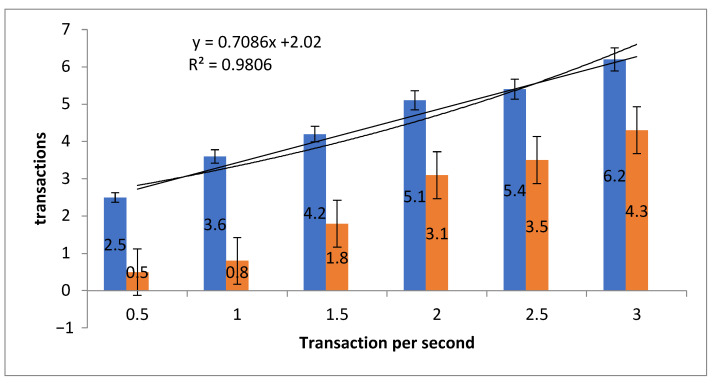
Comparison of transaction speed between PoW and PoS.

A comparison of PoW and PoS is carried out on the simulation and shown in Table 2 and Figure 10. PoW is shown as the blue bars and PoS as the orange bars in the figure.

Table 2 shows a comparison of PoS versus PoW transactions per second. Overall, the present data show that PoS takes less time to validate a transaction, such that, in 0.5 s, PoS validates 2.5 transactions compared with PoW which validates 0.5 transaction or that it takes around twice as long to process the first transaction. The gap closes over longer periods but, on the face of it, the appearance is that PoS is somewhat more time efficient.

(7) $$SE=\frac{\sigma }{\sqrt{n}}$$

A Standard Error (SE) is calculated (Equation (7)) where SE represents the standard deviation of the transactions and total number *n* of transactions per second. The standard deviation shows the variability and dispersion of the transactions. The SE of PoW is ±0.86 and PoS is ±1.54, indicating that the mean value of transactions is close to the actual mean value and that, relatively, the error rate of PoW is less than PoS.

The Straight-line fit and $R^{2}$ are applied to calculate the variation in transactions. The value y=0.7086x shows the difference in time when transactions increase and 2.02 represents the y−intercept. The value of $R^{2}$ at 0.9806 implies a correlation between transaction and time.

**Figure 11:**
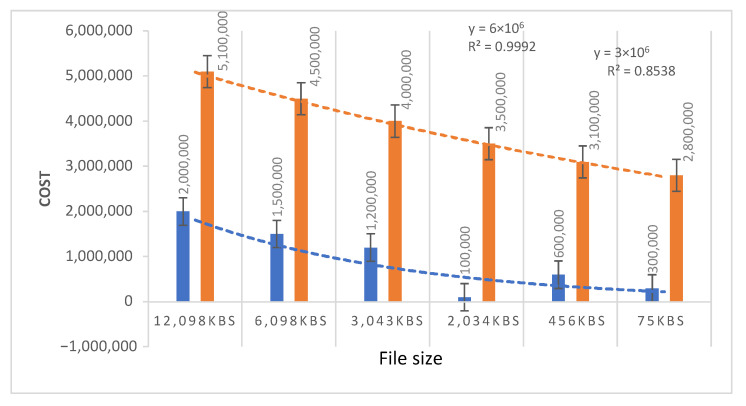
Comparison of smart contract deployment cost of PoW and PoS.

The smart contract deployment cost is set as a default gas price of ten (10) gwei. Various applications of smart contracts consume different amounts of gas. Table 3 and Figure 11 illustrate the gas consumption of the two consensus mechanisms simulated in the system. To calculate the transaction cost and execution cost of PoS and PoW, two algorithms are deployed on the smart contract. The minimum transaction and execution gas of PoS is 3,000,000 with a file size of 75 kb. The minimum transaction gas for PoW is 28,000,000. The experimental analysis shows that PoS is more efficient than PoW in terms of gas consumption on both the transaction and execution of blocks and processing of smart contracts. The PoW requires a lot of computational power to verify blocks and needs significant execution time.

In Figure 11 SE, the straight-line fit and $R^{2}$ are calculated. The SE shows the variability and dispersion of the smart contract deployment cost for PoW and PoS. The SE of PoW is $\pm 46\times 10^{6}$ for file size 12,098 kb; $\pm 41\times 10^{6}$ for 6086 kb; $\pm 35\times 10^{6}$ for 3043 kb; $\pm 32\times 10^{6}$ for 2034 kb; $\pm 27\times 10^{6}$ for 456 kb; and $\pm 23\times 10^{6}$ for 75 kb. The SE of PoS is $\pm 16\times 10^{6}$ for file size 12,098 kb; $\pm 12\times 10^{6}$ is for 6098 kb; $\pm 9\times 10^{6}$ for 3043 kb; $\pm 7\times 10^{6}$ for 2034 kb; $\pm 4.5\times 10^{6}$ for 456 kb; and $\pm 2\times 10^{6}$ for 75 kb. The SE implies that the mean value of deployment cost is close to the actual mean value and that the deployment cost of PoW is relatively high compared with PoS. The straight-line fit (*y*) and $R^{2}$ are calculated to determine the change in deployment cost. The *y* value shows the difference in cost when the file size increases in both PoS and PoW. The $R^{2}$ value shows the correlation between cost and file size.

**Figure 12:**
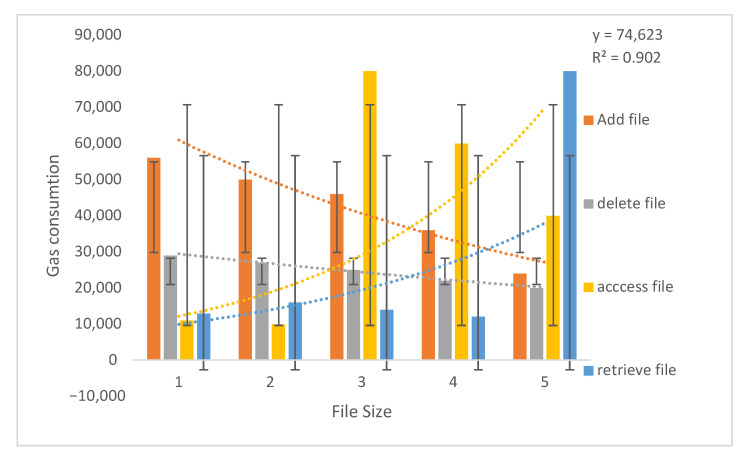
Gas consumption cost to add, delete, access and retrieve files.

The smart contract includes the functions to add, delete, access and retrieve files (Figure 12 and Table 4). The gas is consumed whenever a healthcare provider adds a file to the blockchain network, deletes an existing file from the network with the permission of the record owner, access a file when a patient visits another healthcare provider and retrieves it. Here, the files are prescriptions generated by the healthcare provider and uploaded to the smart contract. Figure 12 and Table 4 present the minimum transaction and execution cost of functions deployed on smart contracts.

The SE calculated for the gas consumption cost of the add function is ±43,432 for 6098 kb; ±38,993 for 3043 kb; ±35,003 for 2034 kb; ±26,321 for 456 kb; and ±15,022 for 75 kb. SE for the delete function is ±24,011 for 6098 kb; ±19,012 for 3043 kb; ±15,001 for 2034 kb; ±14,210 for 456 kb; and ±13,211 for 75 kb. The SE for access functions is ±65,900 for 6098 kb; ±49,324 for 3043 kb; ±33,021 for 2034 kb; ±6003 for 456 kb; and ±5541 for 75 kb. SE for the retrieve function is ±71,122 for 6098 kb; ±12,431 for 3043 kb; ±12,001 for 2034 kb; ±9229 for 456 kb; and ±7671 for 75 kb. SE implies that the mean value of gas consumption cost of functions is close to the actual mean. The straight-line fit is 74,623 and $R^{2}$ is 0.902 calculated as the change in Gas consumption and correlation between Gas and file size.

**Figure 13:**
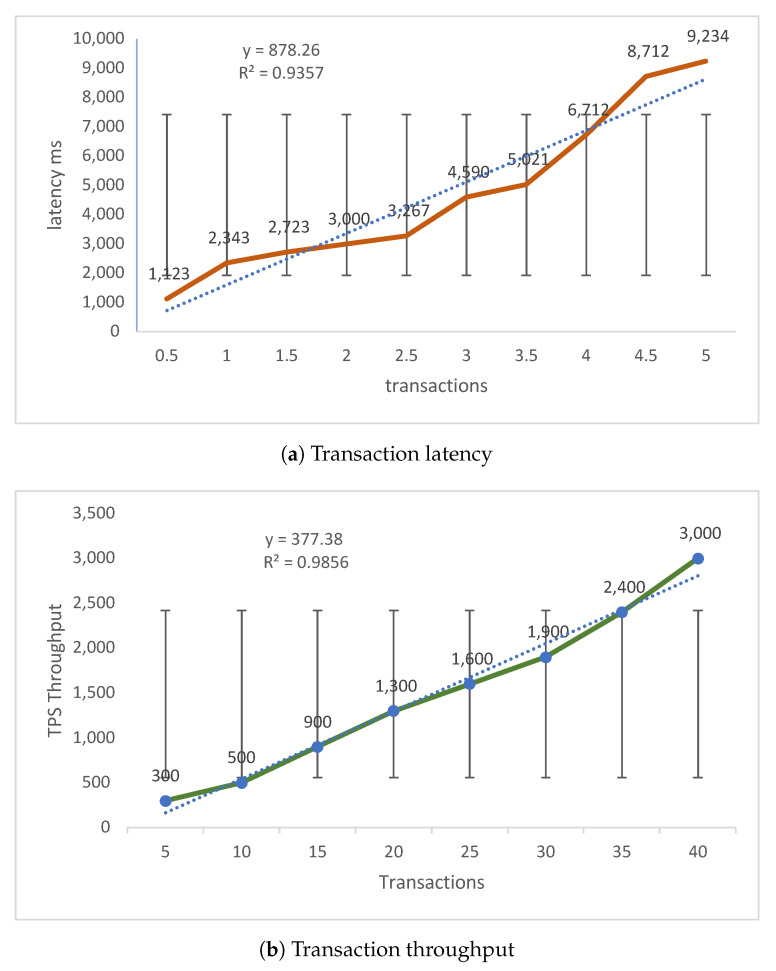
Comparison of transaction latency and throughput.

Figure 13 and Table 5 show a comparison of transaction latency and throughput. Transaction latency (Figure 13a) is how much time it takes for a miner to validate a transaction. The latency is calculated as an average of transactions (Equation (8)) run on the simulated system and measured in milliseconds (ms). The average time to validate transactions is 2343 ms and miners validate five transactions in 9234 ms. The SE calculated for Figure 13a are 0.5 transactions in ±932 ms, 1 in ±1912, 2 in ±2401, 3 in ±3405, 4 in ±4532 and 5 in ±7098. The straight fit line is 878.26, which shows the change in latency, and $R^{2}$ is 0.9357, which shows a correlation between latency and transactions.

(8) $$\mathrm{Latency}=\frac{\mathrm{Total}\;\mathrm{time}}{\mathrm{Total}\;\mathrm{Tx}}$$

Throughput (Figure 13b) is how much time it takes for a transaction be validated and is the average of the total time it takes to process transactions over the overall total of time (Equation (9)).

(9) $$\mathrm{Throughput}=\frac{\mathrm{Total}\;\mathrm{Time}\;\mathrm{tx}}{\mathrm{Total}\;\mathrm{Time}}$$

The simulation data in Figure 9 shows that as the number of users increase, the throughput also increases in a linear fashion. This provides some evidence of the efficiency of the BlockPres system. The SE of transactions over throughput are five transactions in ±300 ms, 15 in ±900 ms, 25 in ±600 ms, 30 in ±1900 ms, 35 in ±2400 ms and 40 in ±3000 ms. The straight fit line is 377.38, which shows the change in throughput, and $R^{2}$ is 0.9856, implying a correlation between throughput and transactions.

**Figure 14:**
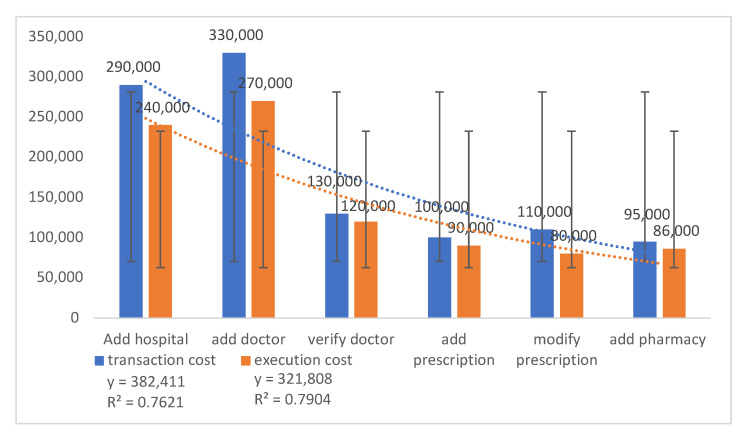
Function cost comparison for transactions and execution.

The transaction and execution cost of adding entities to the smart contract (Table 6 and Figure 14) are calculated as Gwei. The entities are hospitals, doctors, pharmacies and associated functions such as the adding of prescriptions, modification of prescriptions and so on. The cost varies depending on what consensus algorithm is used to perform the smart contract functions. The transaction and execution cost of adding an entity is 260,000, verifying an entity is 125,000, adding and modifying costs are 105,000 and 85,000, respectively, and the costs of adding a pharmacy are 95,000 and 86,000. The SE for the transaction and execution costs of adding a hospital are ±250,000 and ±210,000; costs for adding a doctor are ±280,000 and ±230,000; costs for verifying a doctor are ±100,000 and ±90,000; costs for adding prescription are ±7000 and ±6530; costs for modifying prescription are ±9500 and ±6430; and costs for adding a pharmacy are ±6400 and ±6210. By highlighting the difference between transaction and execution cost, the straight-fit lines for transaction and execution costs are 38.2411 and 32.1808. $R^{2}$ is 0.7621 for the transaction cost and 0.7904 for execution cost, which indicates a correlation between execution cost and transaction cost.

## 10. Conclusions and Future Work

In this paper, a blockchain-based solution is proposed to address issues with patients that experience unequal access to healthcare services. The use of blockchain technology has previously been demonstrated to be of use in HIS. In this study, the solution provides an incentive mechanism to encourage users to engage with health services by using the system and sharing their medical records with healthcare providers. In designing the system, consideration is given to how the principles that underlie blockchain technology can be applied to HIS. From this, BlockPress encourages users to use the system and to receive rewards as tokens.

The DSR methodology is applied for the successful execution of the project: by designing a blockchain-based framework to record the process of prescriptions issued by a healthcare provider, to receive rewards and to provide access to patient records. The healthcare provider and patient can track and authorise transactions during application of public and private keys. Transactions are secured using established cryptographic methods for authentication and authorisation. Moreover, in order to enable critical decisions, the patient obtains control of their data.

An initial evaluation assessed transaction speed and the results demonstrate that this blockchain is, at the very least, suitable for application for BlockPres. Following this, simulations of the model are instantiated on the Ethereum blockchain, which takes advantage of the smart contract and utilises the Solidity language. The simulations are performed using the Remix IDE and Ropsten test network to collect performance data from different consensus mechanisms. The results of the simulations provide promising outcomes.

In the next phases, a BlockPres prototype that utilises the information from this study is being developed. Central to the research direction of this prototype will be to determine the use and application of the incentivisation scheme. In addition, methods for the calculation of incentives that are based on patient input, verification and distribution of incentives between patients, security and privacy of patient accounts and so on will be examined. The prototype will also incorporate IPFS for data storage and retrieval.

## Figures and Tables

**Table 1:** Notations used in the BlockPres framework.

Symbol	Description	Symbol	Description
$HP_{K}$	Healthcare Provider	$PK_{P}$	Patient Public Key
TX	Transactions	$SK_{P}$	Patient Secret Key
$H_{K}$	Hospitals	$PK_{d}$	Doctor Public Key
$Lab_{K}$	Laboratory	$SK_{d}$	Doctor Secret Key
$N_{K}$	Nurse	SC	Smart Contract
$DT_{K}$	Doctor	RC	Registry Contract
$ST_{K}$	Other Staff	H()	Hash function
$PT_{K}$	Patient	$IDT_{P}$	Identity of Patients
$AD_{K}$	Hospital Administration	Kw	Keywords
TS	Time Stamp	*m*	Message
$PH_{K}$	Pharmacy	PRd	Patients record
$GPS_{K}$	General Practitioner Station	*K*	Key
NHI	National Health Index	ADHB	Auckland District Health Board
MOH	Ministry of Health	DB	Database

**Table 2:** Comparison of Transactions per second, PoW vs. PoS.

Transaction	PoS	PoW
Per Second	(Transaction)	(Transaction)
0.5	2.5	0.5
1	3.6	0.8
1.5	4.2	1.8
2	5.1	3.1
2.5	5.4	3.5
3	6.2	4.3

**Table 3:** Comparison of Smart Contract deployment cost of PoS vs. PoW.

File Size (kb)	PoS	PoW
	Gas (mill.)	Gas (mill.)
12,098	2.0	51.0
6098	1.5	45.0
3043	1.2	40.0
2034	1.0	35.0
456	6.0	31.0
75	3.0	28.0

**Table 4:** Gas consumption cost to add, delete, access and retrieve files.

File Size (kb)	Functions (as Gas Consumed)			
	Add File	Delete File	Access File	Retrieve File
6098	56,334	29,110	79,900	79,110
3043	51,023	27,990	58,009	16,012
2034	46,800	25,120	37,100	14,540
456	36,610	22,231	9210	12,203
75	24,022	20,021	7001	10,012

**Table 5:** Transaction latency and throughput.

Latency		Throughput	
**Transactions**	**Latency (ms)**	**Patients**	**Throughput (ms)**
0.5	1123	5	300
1	2343	10	500
2	3000	20	1300
3	4590	25	1600
4	6712	30	2400
5	9234	40	3000

**Table 6:** Function cost comparison for transactions and execution.

Function	Transaction Cost	Execution Cost
Add hospital	290,000	240,000
Add doctor	330,000	270,000
Verify doctor	130,000	120,000
Add prescription	100,000	90,000
Modify Prescription	110,000	80,000
Add pharmacy	95,000	86,000
